# Serological profiling reveals hsa-miR-451a as a possible biomarker of anaphylaxis

**DOI:** 10.1172/jci.insight.156669

**Published:** 2022-04-08

**Authors:** Wojciech Francuzik, Kristijan Pažur, Magdalena Dalke, Sabine Dölle-Bierke, Magda Babina, Margitta Worm

**Affiliations:** Division of Allergy and Immunology, Department of Dermatology, Venereology and Allergology, Charité – Universitätsmedizin Berlin, Corporate Member of Freie Universität Berlin and Humboldt-Universität zu Berlin, Berlin, Germany.

**Keywords:** Immunology, Allergy, Diagnostics

## Abstract

**Background:**

There is a need to support the diagnosis of anaphylaxis by objective markers. miRNAs are promising noncoding RNA species that may serve as serological biomarkers, but their use in diagnosing anaphylaxis has not been systematically studied to our knowledge. We aimed to comprehensively investigate serum biomarker profiles (proteins, lipids, and miRNAs) to support the diagnosis of anaphylaxis.

**Methods:**

Adult patients admitted to the emergency room with a diagnosis of anaphylaxis (<3 hours) were included. Blood samples were taken upon emergency room arrival and 1 month later.

**Results:**

Next-generation sequencing of 18 samples (6 patients with anaphylaxis in both acute and nonacute condition, for 12 total samples, and 6 healthy controls) identified hsa-miR-451a to be elevated during anaphylaxis, which was verified by quantitative real-time PCR in the remaining cohort. The random forest classifier enabled us to classify anaphylaxis with high accuracy using a composite model. We identified tryptase, 9α,11β-PGF2, apolipoprotein A1, and hsa-miR-451a as serological biomarkers of anaphylaxis. These predictors qualified as serological biomarkers individually but performed better in combination.

**Conclusion:**

Unexpectedly, hsa-miR-451a was identified as the most relevant biomarker in our data set. We were also able to distinguish between patients with a history of anaphylaxis and healthy individuals with higher accuracy than any other available model. Future studies will need to verify miRNA biomarker utility in real-life clinical settings.

**Funding:**

This work is funded by the Deutsche Forschungsgemeinschaft (DFG, German Research Foundation) as part of the clinical research unit (CRU339): Food Allergy and Tolerance (FOOD@) (project number 409525714) and a grant to MW (Wo541-16-2, project number 264921598), as well as by FOOD@ project numbers 428094283 and 428447634.

## Graphical abstract

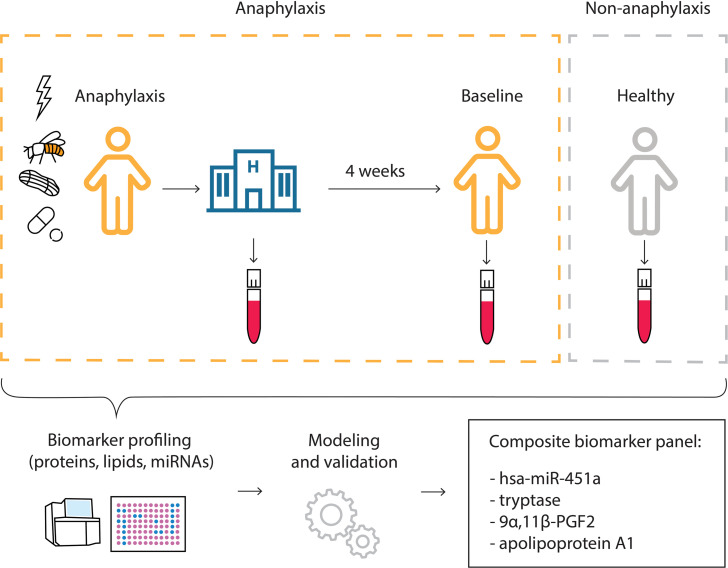

## Introduction

Anaphylaxis is a type I hypersensitivity reaction that is potentially life-threatening if not adequately treated. Although the simultaneous onset of skin symptoms and hypotension (in patients with a history of allergic reactions) suggests an anaphylactic episode, reactions may present atypically and skin symptoms may not be present in up to 20% of patients (1, 2). To more confidently administer adrenaline as the first-line treatment in cases where the elicitor of anaphylaxis is unknown and the diagnosis uncertain, there is a high medical need for objective laboratory parameters supporting the diagnosis of anaphylaxis (preferably using easy-to-acquire biological samples, e.g., blood).

Currently, total mast cell tryptase is the only laboratory parameter routinely used for the diagnosis of anaphylaxis, but its moderate sensitivity and specificity require improvement (3). Moreover, the values of tryptase during acute reaction need to be related to their baseline levels in the same individual because of substantial interindividual variability even at baseline (4). In addition, the relatively late diagnostic window for serum tryptase between 30 and 120 minutes after the onset of first anaphylaxis symptoms is too late given the necessity of rapid treatment initiation. Therefore, multiple efforts have been undertaken in identifying other potential biomarkers of anaphylaxis (5–8).

miRNAs are small noncoding RNAs that regulate gene expression usually by silencing transcription (9). Their use as serological biomarkers has been proposed in various diseases (10). Data on the role of miRNA in human anaphylaxis are largely unavailable. A recent study of food allergies in 4 children reported differential expression of several miRNAs during the reaction, indicating miRNAs’ potential to assist in the diagnosis of anaphylaxis (11). Since several miRNAs were shown to play a role in acute asthma via airway inflammation and hyperresponsiveness (12), we hypothesized that miRNA secretion may also occur in hypersensitivity reactions. The aim of this study was therefore to identify biomarkers predictive of anaphylaxis, with a special emphasis on miRNAs.

## Results

### Study cohort characteristics.

**Figure 1:**
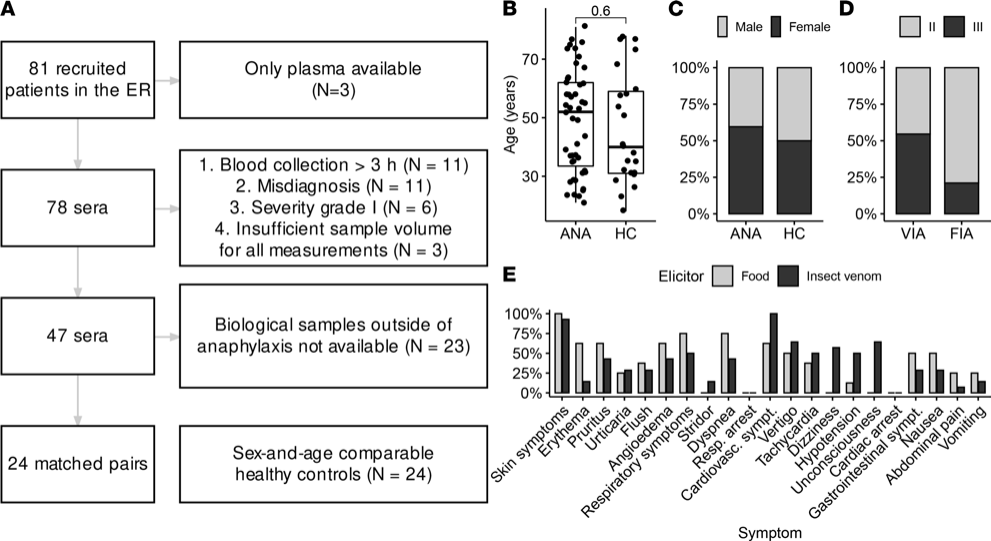
Studied cohort shows clinically relevant reactions. (**A**) Flowchart illustrating the exclusion criteria of the study. (**B** and **C**) Age and sex distribution in the studied cohort and their corresponding controls (ANA, acute anaphylaxis; HC, healthy control; 2-tailed Student’s *t* test, *n* = 71). (**D**) Distribution of elicitors and severity of anaphylaxis according to Ring and Messmer (13) (FIA, food-induced anaphylaxis; VIA, venom-induced anaphylaxis). VIA did not show significantly more severe reactions, classified as Ring and Messmer (13) grade III, than FIA (*P* = 0.089). (**E**) Frequency of symptoms in FIA and VIA.

We prospectively recruited 81 patients from the emergency room with a suspected diagnosis of anaphylaxis. Out of this cohort, 31 patients had to be excluded from the analysis because of low severity, or prolonged time (>3 hours) from the onset of the first symptoms until blood draw in the emergency room, or diagnostic criteria for anaphylaxis not being fulfilled. Second sampling during a diagnostic visit was performed in 24 out of 47 patients with anaphylaxis at the Allergy and Immunology Division (Figure 1A). Twenty-four healthy individuals without a history of hypersensitivity reactions were recruited as controls. The mean age of patients and healthy controls was 48.77 (median = 52) and 46.26 (median = 40) years, respectively, and the sex distribution was comparable (Figure 1, B and C). The majority of patients were White (European 85.11%; 5 or 10.64% were of Middle Eastern descent), and 2 (4.26%) were east Asian. The classification was made by the investigators. The study participants are summarized in Supplemental Table 1 (supplemental material available online with this article; https://doi.org/10.1172/jci.insight.156669DS1).

The majority of cases in our cohort were venom-induced anaphylaxis (VIA, *n* = 22) and food-induced anaphylaxis (FIA, *n* = 19), followed by drugs (*n* = 3) and 3 cases of idiopathic anaphylaxis (Figure 1D). VIA did not show significantly more severe reactions, classified as Ring and Messmer (13) grade III, than FIA (*P* = 0.089). There were no significant differences in severity between sexes (data not shown).

Skin symptoms were most prevalent regardless of elicitor. Symptoms of VIA were more often associated with the cardiovascular system (*P* = 0.036), whereas there was a tendency of greater gastrointestinal and respiratory involvement in FIA than in VIA without reaching significance (*P* = 0.386 and *P* = 0.204, respectively). There were no cases of respiratory or cardiac arrest (Figure 1E).

### Identification of 19 miRNA candidates via next-generation sequencing.

**Figure 2:**
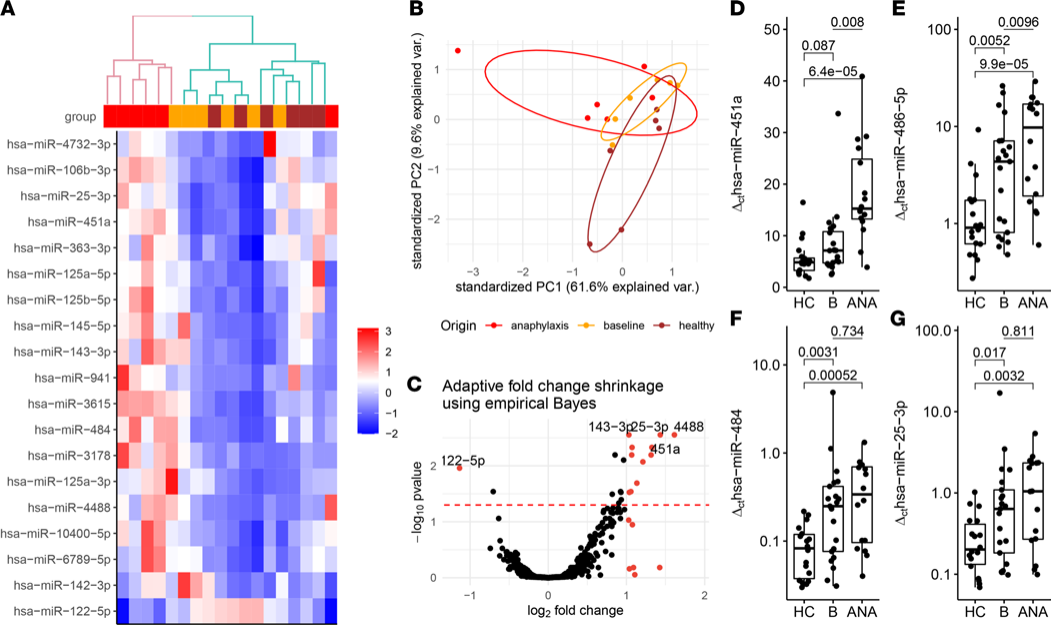
Screening and validation of miRNA candidate biomarkers show different expression in anaphylaxis when compared with healthy individuals and baseline. (**A**) Heatmap of the 19 most differentially expressed miRNAs (using next-generation sequencing) in 6 anaphylaxis samples (top row red) in comparison to the corresponding samples on baseline (top row yellow) applying unsupervised hierarchical clustering with Euclidean distances. Additional 6 healthy control samples (top row brown) were provided for completeness. (**B**) Principal component analysis of the sequenced cohort in corresponding colors (the 50 most differentially expressed miRNAs; data scaled and centered). (**C**) Volcano plot illustrating differentially expressed miRNA upon anaphylaxis (*n* = 6) compared with baseline (*n* = 6). Fold changes were adjusted using the Ashr algorithm. Red dots mark miRNAs with adjusted log-fold change greater than 1 and less than –1. Red dashed line indicates adjusted *P* < 0.05 (Wald test with Benjamini-Hochberg FDR, *n* = 2656 miRNAs per sample). (**D**–**G**) Quantification of selected miRNAs in serum using real-time qPCR on sera from patients with anaphylaxis in acute (ANA, *n* = 16), baseline (*n* = 16), and healthy controls (HC, *n* = 20). Comparisons for HC versus baseline and HC versus ANA; 2-tailed Student’s *t* test for unpaired data with Holm-Šidák correction. Comparisons between baseline and ANA; 2-tailed Student’s *t* test for paired data.

The initial screening of 6 patients with anaphylaxis (3 VIA and 3 FIA) resulted in successful labeling and counting of 2656 miRNAs after removing entities with fewer than 100 counts. We observed differential expression of 19 miRNAs, with *P* values less than 0.05 after FDR adjustment using Benjamini-Hochberg correction, between samples taken from the patients in anaphylaxis and their respective baselines (Figure 2A). The samples from an acute reaction clustered together when unsupervised clustering was performed, separating them from healthy and baseline as indicated in the dendrogram (Figure 2A).

Principal component analysis showed some but no precise differentiation across the groups (regardless of the number of differentially expressed miRNAs, Figure 2B). Based on the differential expression analysis (with the DESeq2 package for R; ref. 14; Figure 2C), we selected 5 miRNAs as potential candidate biomarkers for further study: hsa-miR-451a, hsa-miR-143-3p, hsa-miR-486-5p, hsa-miR-25-3p, and hsa-miR-484, performing quantitative real-time PCR (RT-qPCR) validation on the sera of patients who provided baseline samples.

hsa-miR-451a and hsa-miR-486-5p showed a significant difference in values between groups, which was highly enhanced in anaphylaxis samples (Figure 2, D and E). Notably, a control group of patients with atopic dermatitis and no medical history of anaphylaxis showed similar levels of hsa-miR-451a to healthy controls (Supplemental Figure 6). Conversely, hsa-miR-25-3p and hsa-miR-484 showed significantly higher values (Figure 2, F and G) in both anaphylaxis and baseline samples when compared with healthy controls but did not differ between anaphylaxis and baseline samples of the same patients. hsa-miR-143-3p did not show significant differences across the groups (Supplemental Figure 1).

### Patterns of serological biomarkers in anaphylaxis — a collective view.

**Figure 3:**
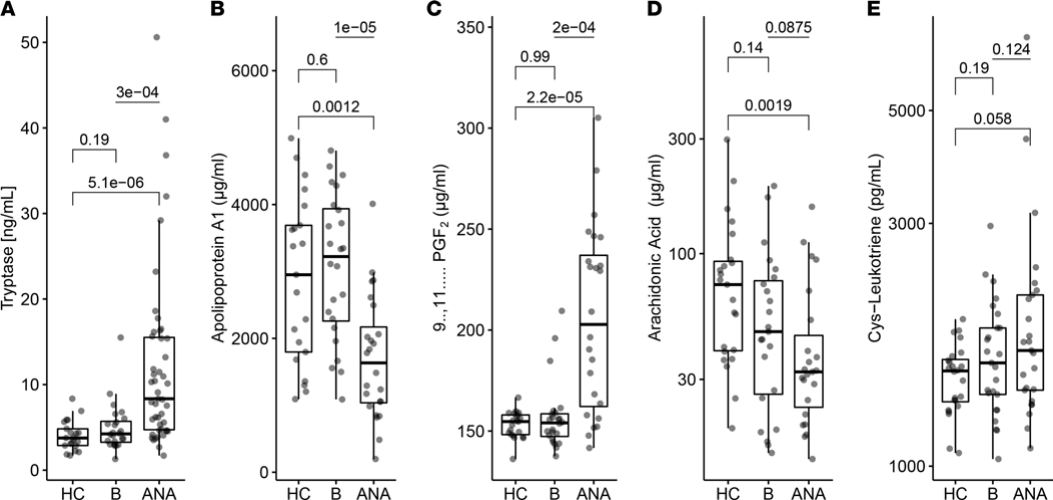
Protein and lipid candidate serum biomarkers differentiate between anaphylaxis and baseline. (**A**) ImmunoCAP measurement of tryptase in serum. (**B**–**E**) ELISA measurement of selected biomarkers in healthy controls (HC), baseline, and acute anaphylaxis (ANA). Comparisons for HC (*n* = 24) versus baseline (*n* = 24) and HC versus ANA (*n* = 24); 2-tailed Student’s *t* test for unpaired data with Holm-Šidák correction. Comparisons between baseline and ANA; 2-tailed Student’s *t* test for paired data.

We observed elevated tryptase in anaphylaxis compared with baseline and healthy control samples (Figure 3). Moreover, we were able to confirm our recently identified markers 11β-prostaglandin F2α (PGF2, increase) and apolipoprotein A1 (ApoA1, decrease) in this independent cohort (6, 8). In addition, we identified arachidonic acid (AA) to be significantly decreased in our anaphylaxis samples when compared with healthy controls (Figure 3D). No significant differences were observed for other potentially relevant proteins, including cysteinyl leukotriene (cys-LT; Figure 3E), CCL13, CCL27, apolipoprotein E (ApoE), CCL17, eosinophil cationic protein, or chitinase-3-like protein 1 (YKL-40), as shown in Supplemental Figure 1, A–F.

To better understand the biomarker profiles during anaphylaxis, we stratified the patients according to the elicitor and grade of reaction. Ring and Messmer grade III reactions mostly showed greater differences in the measured biomarkers when compared with healthy controls and baseline (Supplemental Figure 2A). Biomarker levels were not altered by adrenaline therapy, except for AA, which was lower in patients who did not receive adrenaline (Supplemental Figure 2B).

hsa-miR-451a was higher in patients undergoing VIA when compared with FIA (Supplemental Figure 2C). Baseline levels of hsa-miR-25-3p were elevated in VIA both at baseline and during anaphylaxis when compared with FIA. YKL-40 and AA showed the expected trends in VIA but not in FIA, despite comparable levels in the baseline of both groups (Supplemental Figure 2C). Levels of hsa-miR-25-3p were elevated at baseline and anaphylaxis in VIA when compared with FIA.

Cross-sectional time analysis of the biomarker levels in various time intervals from the studied cohort indicated that tryptase and PGF2 levels peaked (and ApoA1 bottomed) between 60 and 100 minutes after anaphylaxis onset. The hsa-miR451a showed a sideways trend in time, with the highest value observed at around 60 minutes. AA levels after anaphylaxis were lower in cases when the time interval from anaphylaxis onset to blood draw was extended. The highest levels of hsa-miR-25-3p were observed 120 minutes after the onset of anaphylaxis symptoms (Supplemental Figure 3, A–F).

### Random forest accurately classified samples using a set of biomarkers.

**Figure 4:**
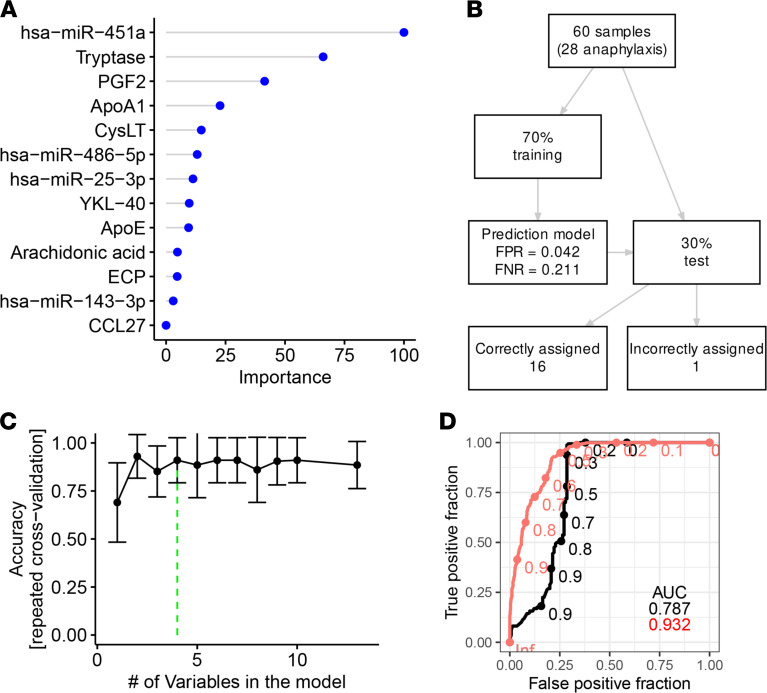
Serological biomarkers allow prediction of an acute anaphylactic episode using a random forest classification model. (**A**) Feature importance score using a random forest algorithm with all potential biomarkers of anaphylaxis. (**B**) Summary of sample selection for the algorithm training and test sets. (**C**) Automatic selection of best features out of the 18 initial ones reported in **A** using recursive feature elimination algorithm (15). (**D**) Receiver operating characteristic (ROC) in a random forest model with 4 top predictors (hsa-miR-451a, tryptase, PGF2, and apolipoprotein A1 — red curve) compared with a model including tryptase, apolipoprotein A1, and PGF2 — black curve.

To assess the diagnostic value of the distinct biomarkers, we performed a classification of samples using the random forest algorithm. In the initial model, all candidates were supplied as predictors and the importance of each predictor was calculated, indicating hsa-miR-451a as the most important predictor (Figure 4A). To minimize the set of predictors, we performed recursive feature elimination and arrived at a model with 4 predictors having the highest accuracy (Figure 4C). Hyperparameter tweaking optimally indicated 2 randomly selected variables for each split (Supplemental Figure 4B). We performed 10 runs of 10-fold repeated cross-validation using the caret package for R (15) and arrived at 0.93 area under the receiver operating characteristic (ROC) curve for the top 4 predictors (out-of-bag error = 12%, Supplemental Figure 4A). This was 0.15 higher when compared with a model where random forest used 3 predictors for classification: tryptase, PGF2, and ApoA1. Thus, the addition of hsa-miR-451a increased the precision to detect an acute reaction (Figure 4D).

To validate the model, we used the remaining 30% of randomly preselected observations (*n* = 9 during anaphylaxis; *n* = 8 on baseline). The final model correctly classified 16 cases (94.12%). Only 1 sample originating from the baseline group was incorrectly assigned as anaphylaxis (Figure 4B).

### Enrichment analysis of the differentially expressed miRNAs.

**Figure 5:**
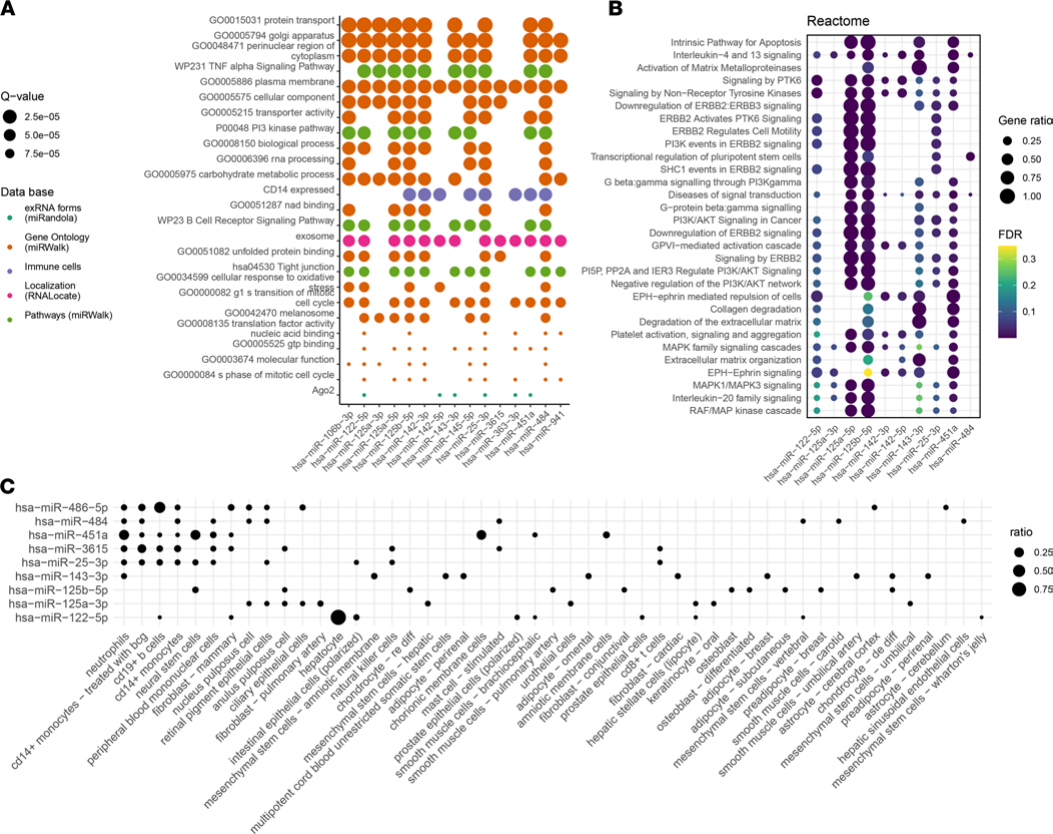
miRNA enrichment and localization analysis indicate the role of the most differentially expressed miRNAs in protein transport and cell signaling processes in immune cells. (**A**) Enrichment analysis on the miRNA set produced by differential expression analysis using Enrichment Analysis and Annotation Package 2.0 (miEAA2.0). The first most differentially expressed miRNAs visualized according to gene set membership in the 25 most relevant gene sets (*q* value < 0.005; *P* value adjusted using Benjamini-Hochberg FDR). (**B**) Result of miRNA enrichment analysis using MIENTURNET (17). Functional enrichment using the Reactome pathways (21). (**C**) Cell-specific expression of miRNA in FANTOM5 mammalian short RNA expression atlas (18).

We used 2 computational tools (miEAA2.0, ref. 16; MIENTURNET, ref. 17) and FANTOM5 mammalian expression miRNA atlas (18) to predict the pathomechanistic relevance of selected miRNAs in anaphylaxis. The miEAA2.0 analysis yielded 163 enriched subcategories (out of over 13,000) in 7 categories out of 19 used. Figure 5A summarizes the first 25 most significantly enriched subcategories.

Most of the enriched subcategories came from the Gene Ontology (miRWalk) category, indicating enrichment in protein transport, carbohydrate metabolism, oxidative stress, inflammation through the TNF-α signaling pathway, and cell activation with the PI3 kinase pathway. Regarding the localization of miRNAs based on the RNALocate2.0 database (19), we saw enrichment in the exosome subcategory, as expected.

Concurrent analysis was performed using the MIENTURNET online tool (17) with 19 differentially expressed miRNAs (2 minimum miRNAs per gene target and FDR < 0.05) using the miRTarBase database (20). Target prediction indicated highly significant involvement of trans-Golgi network proteins (Golgin A8 A and B, vacuolar protein sorting-associated protein) as well as signaling (protein phosphatase 1 regulatory subunit 37), carbohydrate metabolic processes (hexokinase 2), and transcription regulatory processes (zinc finger protein 264, RNA binding motif protein 27) (Supplemental Figure 5). Target genes identified in miRTarBase overlapped with the pathways identified by miEAA2.0 analysis.

Reactome (21) pathway analysis with the predicted miRNA target genes identified enrichment in apoptosis and IL-4 and IL-13 signaling, activation of metalloproteases, and multiple tyrosine kinase signaling (Figure 5B).

The cellular origin of the 12 most differentially expressed miRNAs was determined based on the data from the FANTOM5 Project (18), providing expression levels of known miRNAs in 118 different cell types. No expression of hsa-miR-3178 or hsa-miR-10400-5p was detected. Neutrophils, monocytes, and B cells were the primary source of the most differentially expressed miRNAs, and hsa-miR-451a was primarily expressed in neutrophils (Figure 5C).

### miRNAs identified patients prone to anaphylaxis.

Parameters that distinguish patients at risk of developing anaphylaxis remain unknown but are highly desirable for risk assessment and the implementation of prophylactic measures or even for retrospective diagnosis.

**Figure 6:**
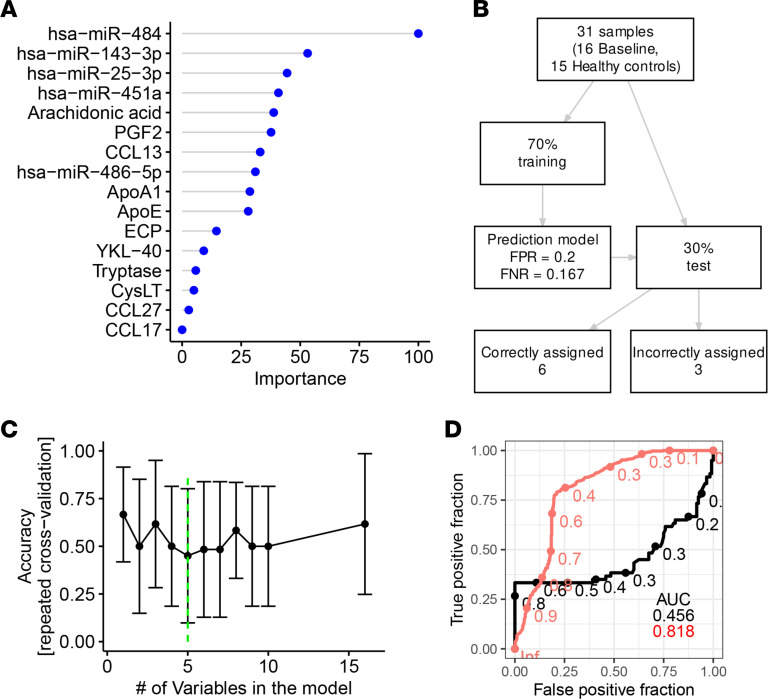
Serological biomarkers show potential to predict patients at risk of anaphylaxis. (**A**) Feature importance score using a random forest algorithm with all potential biomarkers. (**B**) Flowchart for the modeling procedure, including model evaluation using repeated cross-validation. FPR, false positive rate; FNR, false negative rate. (**C**) Automatic selection of best features out of th e 17 initial ones reported in **A** using recursive feature elimination algorithm. (**D**) ROC AUC in a random forest model with 5 predictors (hsa-miR-484, hsa-miR-25-3p, hsa-miR-451a, apolipoprotein E, and YKL-40 — red curve) compared with a model including tryptase, apolipoprotein A1, and PGF2 — black curve.

In the preceding data (Figure 6), we fortuitously observed that in addition to the steep increase in hsa-miR-451a during anaphylaxis, other entities were enhanced in baseline measurements vis-à-vis healthy controls. Since this provided a strong hint that miRNAs may serve in the identification of patients at risk, we employed the same procedure as above to examine this possibility. This was performed by designing a random forest model. Indeed, the combination of 5 predictors (hsa-miR-484, hsa-miR-25-3p, hsa-miR-451a, ApoE, and YKL-40) resulted in a random forest model showing good accuracy to differentiate between patients prone to anaphylaxis (i.e., between baseline samples from patients who had anaphylaxis approximately 4 weeks prior to sampling and samples from healthy controls) (Figure 6, A and C). The model was greatly improved against the reference model (tryptase, PGF2, ApoA1) with an area under ROC curve of 0.82 versus 0.46, respectively (0.36 difference, Figure 6D).

Though the predictive capacity of the model was somewhat limited because of the lower number of observations used for training (*n* = 22, out-of-bag error = 18.18%), the model still correctly assigned 66.67% of cases (Figure 6B) on the 30% of previously unseen data. Although more data are needed, this is arguably the first successful step toward the detection of a biomarker panel to assist in the identification of patients prone to anaphylaxis.

## Discussion

This study found hsa-miR-451a to be a robust biomarker of anaphylaxis, which may improve diagnostic accuracy, especially when combined with other available biomarkers. In our prospective clinical observational study, we found higher expression of hsa-miR-451a in the serum of patients with anaphylaxis compared with healthy controls and nonacute sera and were able to model its diagnostic capacity using a random forest classifier. Importantly, we achieved high classification accuracy when the model was tested on previously unseen real-life data.

The studied cohort adequately represented anaphylaxis cases in Europe. Insect venom was the most prevalent elicitor of anaphylaxis in our cohort, corresponding to the previously published data for Europe (22). Although US epidemiological data indicate medication followed by food as the primary triggers of anaphylaxis (23), food anaphylaxis is most prevalent in children (24), who were not included in this study. In accordance with our data, the clinical presentation of anaphylaxis with cardiovascular symptoms is known to be more prevalent in VIA than in FIA (1, 25), and the frequency of grade IV cases (Ring and Messmer scale) is known to be low (26). We did not include cases with Ring and Messmer grade I, as they did not meet the definition of anaphylaxis by National Institute of Allergy and Infectious Disease/Food Allergy and Anaphylaxis Network (NIAID/FAAN) (27). In line with previous reports, we observed more severe cases in VIA (1). One of the limitations of this study was the lack of anaphylaxis due to drugs (we had only 1 paired case). Therefore, this may be of interest for future studies.

Nevertheless, acquiring samples from patients in anaphylaxis is difficult because the main concern is to provide immediate therapy to a patient with a potentially lethal allergic reaction. Therefore, in some cases, a low amount or incorrect biomaterial was sampled upon the emergency room visit. Fortunately, a substantial number of patients complied with a follow-up visit to our Allergy and Immunology Division approximately 4 weeks after the reaction so that patient-matched serum samples during anaphylaxis and baseline were available for the study. This was not possible with 48% of patients, yet we were able to use unpaired samples for the random forest modeling, which allowed us to achieve good model accuracy. It needs to be pointed out that in real-life scenarios, patient-matched baseline sera are unavailable in most situations, so biomarker models that do not require the matched nonacute serum from patients will best serve in the clinic.

This study explored a molecular category rarely investigated in the context of anaphylaxis, i.e., miRNAs, and comprehensively assessed them in regard to other high-confidence biomarkers to grade their utility. The selection of protein and lipid biomarkers was made based on literature research and our findings.

Tryptase has been extensively studied as a biomarker of anaphylaxis. The gold standard involves the measurement of serum total tryptase during an acute phase followed by a baseline measurement (≥24 hours; ref. 28). Although tryptase levels correlate with the severity of anaphylaxis (29) and this is also visible in our data, 2 similarly designed studies conducted in emergency departments failed to confirm a predictive role of tryptase or histamine in the diagnosis of anaphylaxis (30, 31). Nonetheless, tryptase is the best-studied entity already in clinical use despite its limitations. However, we previously identified potentially new candidates, demonstrating the predictive power of PGF2, cys-LTs, ApoA1, and ApoE in the diagnosis of anaphylaxis (6, 8). Notably, PGF2 and ApoA1 were not only reproduced in our current, broader study using an independent cohort, but they also turned out to be in the top 4 classifiers of the random forest, and thereby part of the best biomarker composite model to predict anaphylaxis. AA was selected because it is a precursor for PGF2 synthesis (32). Upon anaphylaxis, levels of AA were expected to decrease as it is metabolized to prostaglandins, leukotrienes, and thromboxanes, and this was indeed observed. The other candidates included were mainly based on their utility in other atopic or mast cell–driven diseases. For example, CCL27 (CTAK) is secreted in the skin and seems to be associated with urticaria (33). Although skin symptoms were highly prevalent during anaphylaxis in our cohort, there were only a few samples with an increase in CCL27 in serum. CCL13 (MCP-4) has been implicated in inflammatory processes in asthma (34, 35), and CCL17 (TARC) is a biomarker of atopic dermatitis severity with a reported case of transient increase after FIA (36). YKL-40 has been implicated in atopic diseases (allergic rhinitis, ref. 37; asthma, refs. 38, 39; and food allergy, ref. 40). Raised eosinophil cationic protein was described in food anaphylaxis (41) and food provocation (42). However, none of them qualified as a potential biomarker of anaphylaxis in our study.

ApoA1 was discovered through a proteomics screen in our previous study (8). Since the strategy proved successful in biomarker exploration, we reapplied an analogous unbiased approach to exploit the untapped potential of miRNAs. The use of paired biosamples (during anaphylaxis and baseline) allowed us to achieve decent group discrimination in unsupervised hierarchical clustering, despite the relatively low number of sequenced samples. By using stringent differential expression analysis and state-of-the-art tools (14, 43), we were able to identify 19 significantly expressed miRNAs, and we extensively tested 5 promising candidates by RT-qPCR in the whole cohort. The next-generation sequencing data could be reproduced with a larger cohort, indicating the usefulness of this approach for the identification of serological biomarkers.

During modeling, we used a reference random forest model with 3 predictors that have previously shown predictive potential in diagnosing anaphylaxis. In our previous publication, a composite linear model using ApoA1 and PGF2 (8) was able to discriminate between anaphylaxis cases and healthy controls (but the results were not cross-validated). Therefore, we benchmarked our modeling to a presumably best-performing composite model using ApoaA1, tryptase, and PGF2. The addition of hsa-miR-451a significantly improved the model accuracy and proved valid upon testing with previously unseen data. Notably, hsa-miR-451a was more important as a predictor for the random forest classifier than tryptase.

Regarding cellular origin, hsa-miR-451a was identified to be expressed by neutrophils and monocytes based on the differentially expressed miRNAs in FANTOM5 (18). In addition, hsa-miR-451a is known to be expressed by erythrocytes (44–47), which were not included in the FANTOM5 cell atlas. Based on that, the increase in serum levels of hsa-miR-451a might be a) coreleased into serum along with cellular activation and degranulation processes; b) linked to the degradation or release from erythrocytes upon anaphylaxis. Future studies will have to address the major cellular sources operative in anaphylaxis and mechanisms of release (direct versus effector cell driven, e.g., via mast cells or basophil activation). Although we demonstrated that hsa-miR-451a was not increased in other atopic diseases (atopic dermatitis), it would be crucial to verify its specificity when compared with other shock syndromes (i.e., myocardial infarction, sepsis, and hypotensive shock).

An intriguing and unexpected outcome of this study was the differential miRNA profile in patients prone to anaphylaxis (patients with anaphylaxis sampled during baseline) versus healthy individuals. Not all individuals with an increased specific IgE (sIgE) to a particular allergen respond to the allergen in question, and the severity of the reaction and organ involvement are likewise unpredictable by sIgE levels or other diagnostic tests, such as a skin prick test or basophil activation test (48). Most importantly, the use of skin prick tests or sIgE failed to be predictive of reaction severity in children allergic to certain foods (49). Currently, the only usable predictors of future anaphylaxis severity are clinical symptoms of the previous reaction and the presence of the cofactors of anaphylaxis (50). The European Guidelines for the Management of Anaphylaxis (PRACTALL) underline the need for an objective, predictive biomarker of future reaction severity (51). At the moment, there are no point-of-care tests available for anaphylaxis, and the first step toward achieving their creation is to confirm the value of miRNAs as a diagnostic tool in a clinical setting. With the rapid development in nucleic acid tests seen during the COVID-19 pandemic (52), we can expect increasing use of similar technology for the diagnosis of other diseases.

It is therefore a major unmet clinical need to identify patients who are at risk of severe hypersensitivity reactions based on serum biomarkers alone. This knowledge can be used to increase patients’ awareness, provide additional prophylactic measures, and even help in the differential diagnosis of anaphylaxis retrospectively using serum. So far, no single or composite biomarkers are available to meet this aim. Here, we found that our proposed composite biomarker (hsa-miR484, hsa-miR-25-3p, hsa-miR-451a, ApoE, and YKL-40) could distinguish between patients prone to anaphylaxis and healthy individuals with a higher accuracy than any other available model. Interestingly, the miRNAs that distinguish patients prone to anaphylaxis from healthy individuals were different than the aforementioned hsa-miR-451a, the latter best suited to diagnose acute symptom precipitation. This was further emphasized by random forest analysis whereby the most significant predictors of the 2 independent questions (i.e., to diagnose acute anaphylaxis and to identify patients at risk) barely overlapped (compare Figure 4 with Figure 6). In fact, of all biomarkers measured, only miRNAs turned out to be reasonable predictors of anaphylaxis risk, highlighting the major potential of this class of biomolecules. Thus, the results can serve as a basis to implement miRNAs in the analytic makeup to diagnose an acute anaphylaxis attack. In addition, they provide a rationale for future research to prospectively identify patients at risk based on serological miRNA profiles alone.

## Methods

### Inclusion criteria, sample collection, and storage.

We obtained serum samples from adult patients undergoing anaphylaxis who were admitted to the emergency room and provided written informed consent, as well as control (baseline) serum samples at least 1 month after anaphylaxis. The diagnosis of anaphylaxis was made according to the NIAID/FAAN criteria (27). We also acquired serum samples from healthy individuals who did not report a history of allergic diseases as well as a second control group consisting of patients with atopic dermatitis without previous medical history of anaphylaxis. After collecting the whole blood, it was left undisturbed at room temperature for 20 to 30 minutes to allow blood to clot. Samples were then centrifuged in a prechilled centrifuge with a horizontal rotor (swing-out head) for 5 minutes at 1500*g* at 4°C, aliquoted, and stored at –80°C.

### Protein and lipid biomarker measurement.

Human serum samples were analyzed using ELISA kits provided by R&D Systems (human CCL17, DY364; human CCL13, DY327; human CCL27, DY376; human YKL-40, DC3L10), Cusabio Technology (human AA, CSB-E09040h; human eosinophilic cationic protein, CSB-E11729h), Cayman Chemical Company (11β-prostaglandin F2α, 516521; cysteinyl leukotriene, 500390-96), Abcam (human ApoA1, ab108804), and Raybiotech Life (human ApoE, ELH-ApoE) following the manufacturers’ protocols. The total concentration of tryptase in serum was measured by ImmunoCAP (Thermo Fisher Scientific).

### Serum miRNA extraction and profiling by next-generation sequencing.

Circulating miRNAs were isolated from 200 μL serum by the miRNeasy serum/plasma kit (Qiagen, 217184) and quantified with the Agilent 2100 Bioanalyzer system. Library preparation was performed using the QIASeq miRNA library kit (Qiagen, 331502) according to the manufacturer’s instructions. Briefly, a total of 5 μL RNA was used for miRNA library preparation. After ligation of the RNA 3′ and RNA 5′ adapter, universal cDNA synthesis with unique molecular identifier assignment, cDNA cleanup, library amplification, and library cleanup were performed. Library preparation quality control was performed using TapeStation (Agilent Technologies).

Sequencing was performed on the Illumina miSeq platform. Samples from 6 patients undergoing anaphylaxis were compared with their corresponding sera taken outside of an allergic reaction (i.e., baseline). The obtained reads were initially trimmed with cutadapt version 2.4 (53) using the first 10 bases of the Qiagen 3′ adapter sequence. Reads shorter than 10 bases after trimming were removed. Over 96% of reads in each sample contained an adapter. Reads were aligned to the mirBase (release 22.1) human miRNA sequences (54) and counted using the Mirdeep2 package (55) (v. 2.0.1.2). To this end, reads were trimmed as described above, converted to the FASTA format, and collapsed using the collapse_reads_md.pl program. Quantification was performed with the quantifier.pl program. Quality control was performed using fastQC v. 0.11.8 (56), RNA-Seqc, and dupRadar (57).

### Differential expression analysis.

Differential expression of miRNA sequencing data from 6 paired samples was analyzed using the DESeq2 package (14) for R (58), with log-fold change shrinkage using the Ashr algorithm (59). Stringent FDR adjustment was applied for the *P* values (60). A heatmap of differentially expressed miRNA was produced using the heatmaply package (61), with row-wise scaling and automatic clustering using Euclidean distances.

### Validation of miRNA using RT-qPCR.

The differentially expressed miRNAs were further validated using reverse transcription RT-qPCR. Briefly, total RNA was isolated from 200 μL serum by the miRNeasy serum/plasma kit (Qiagen, 217184) according to the manufacturer’s instructions. In addition, 3.5 μL miRNeasy serum/plasma spike-in control (Qiagen, 219610) at 1.6 × 10^8^ copies/μL was added to each sample. The total RNA was reverse-transcribed using miRCURY LNA RNA kit (Qiagen, 339340) that generates universal cDNA templates for all miRNAs present in the sample. The synthetic spike-in (UniSp6, Qiagen, 339340) was added to each sample, and the reaction was performed in the GeneTouch thermal cycle (Bioer). Then, miRNA-specific quantification was performed using miRCURY LNA SYBR Green kit (Qiagen, 339347) according to the manufacturer’s instructions. The expression of target miRNAs was normalized to the cel-miR-39-3p synthetic spike-in added during total RNA extraction.

### Classification model.

The supervised machine learning classification model was done using a random forest algorithm with the help of the caret package (15). All samples with complete observations (without missing values) were included in the training set and divided into anaphylaxis and baseline groups (the latter contained healthy controls and patients’ baseline samples). Features for the final random forest model were selected by a recursive feature elimination algorithm on the set of initially available biomarkers. Variable importance was derived from the initial random forest model, which included all predictors. Hyperparameter optimization was internally performed by the caret package to identify the optimal number of randomly drawn candidate variables at each decision tree split, based on the out-of-bag error estimate (62). The model fitness was calculated using 10 sets of 10-fold repeated cross-validation.

### Target gene prediction and functional enrichment.

The functional miRNA set enrichment analysis was performed using the miEAA2.0 online tool (16). The following steps were performed: a) An ordered set of the 500 most differentially expressed miRNAs based on the output from DESeq2 (adjusted *P* values of the 6 screened patients with anaphylaxis and their corresponding baseline samples) was provided for the miEAA2.0. b) Subsequently, the algorithm cross-referenced known and predicted miRNA-gene interaction targets, restricting the significance level to 0.005 and the minimum required miRNA hits per subcategory of 5. We used FDR (Benjamini-Hochberg) *P* value adjustment for the whole set of analyses in all categories. c) The resulting miRNA list for miRNA Set Enrichment Analysis (miRSEA) in all available 19 default gene-set categories included miRTarBase and miRWalk (for gene ontology), cell type–specific atlas, immune cell gene sets, localization (based on RNALocate), and pathways (based on miRWalk database). The 25 most significantly enriched sets were subsequently plotted to indicate the presence (or lack thereof) of the 13 most significantly differentially expressed miRNAs in each of the gene sets.

Additionally, a set of the 19 most differentially expressed miRNAs was provided for the MIENTURNET (17) online tool. After predicting the gene targets using miRTarBase 8.0 (20), the resulting gene sets were used in an enrichment analysis using the Reactome database (21) with 2 intersections.

Data from the FANTOM5 Project promoter level mammalian expression atlas (18) were used to determine the cellular origin of the most differentially expressed miRNAs. Relative abundance was calculated as a ratio of each candidate miRNA expression in a specific cell type (tags per million) to the global expression level in all characterized cells.

### Data availability.

The sequencing data presented in this study have been deposited in the European Nucleotide Archive under accession number PRJEB50710.

### Statistics.

A 2-tailed Student’s *t* test was used for comparing normally distributed values between unpaired observations, with Holm-Šidák *P* value correction for multiple comparisons. Paired data were analyzed using a paired 2-tailed Student’s *t* test with Holm-Šidák correction where appropriate. Next-generation sequencing–derived data were analyzed using a Wald test with Benjamini-Hochberg FDR to correct for multiple comparisons. The box plots in the figures depict the minimum and maximum values (whiskers), the upper and lower quartiles, and the median. The length of the box represents the interquartile range. *P* values less than 0.05 were considered significant.

### Study approval.

The IRB of the Charité Universitätsmedizin Berlin approved this study (EA1/079/06). Written informed consent was acquired from individuals prior to participation in this study.

## Author contributions

WF wrote the initial manuscript, analyzed the data, and designed the model and the computational framework. KP wrote the initial manuscript, conceived and performed the experiments, and analyzed the data. (WF and KP contributed equally to this work; names are in alphabetical order.) MD acquired the clinical samples, verified the analytical methods, performed the experiments, and critically revised the manuscript. SDB contributed to the design and implementation of the research and the analysis of the results and critically revised the manuscript. MB conceived the analytical approach, developed the theoretical framework, supervised the findings of this work, and wrote the manuscript. MW conceived of the presented study, supervised the clinical aspect of the study, developed the theoretical framework, aided in interpreting the results, and critically revised the manuscript.

## Supplementary Material

Supplemental data

ICMJE disclosure forms

## Version 1 02/24/2022

In-Press Preview

## Version 2 04/08/2022

Electronic publication

## Version 3 04/26/2022

Additional acknowledgment for funding received for publication costs.
