# Supplementary material for: Serological profiling reveals hsa-miR-451a as a possible biomarker of anaphylaxis
Source: JCI Insight. 2022 Apr 8;7(7):e156669. doi: 10.1172/jci.insight.156669 (PMC9057591; doi:10.1172/jci.insight.156669)
Supplement: Supplemental data [file jciinsight-7-156669-s282.pdf]

Suplementary Figures for online repository

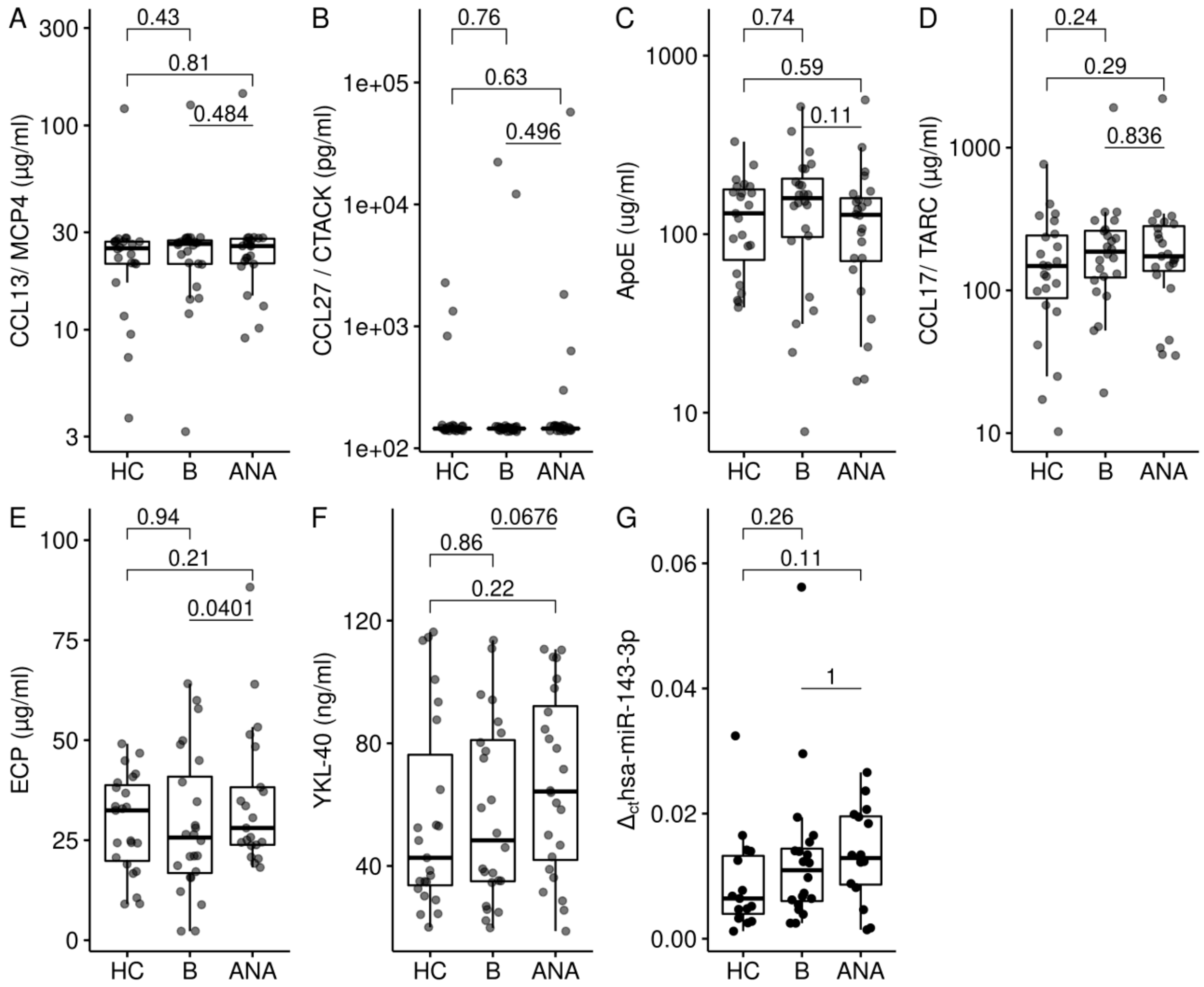

Figure S1: ***Candidate biomarkers without specificity to detect anaphylaxis.*** *A – F) Protein candidates measured by ELISA, differences between healthy (HC, n = 24), and anaphylaxis patients outside (B, n = 24) and during anaphylaxis (ANA, n = 24). G) miRNA delta Ct values (compared to spike in) measured by RT-qPCR in three groups. HC vs. B and HC vs. ANA - two-tailed Student's T test; B vs. ANA - two-tailed Student's T test for paired data.*

8

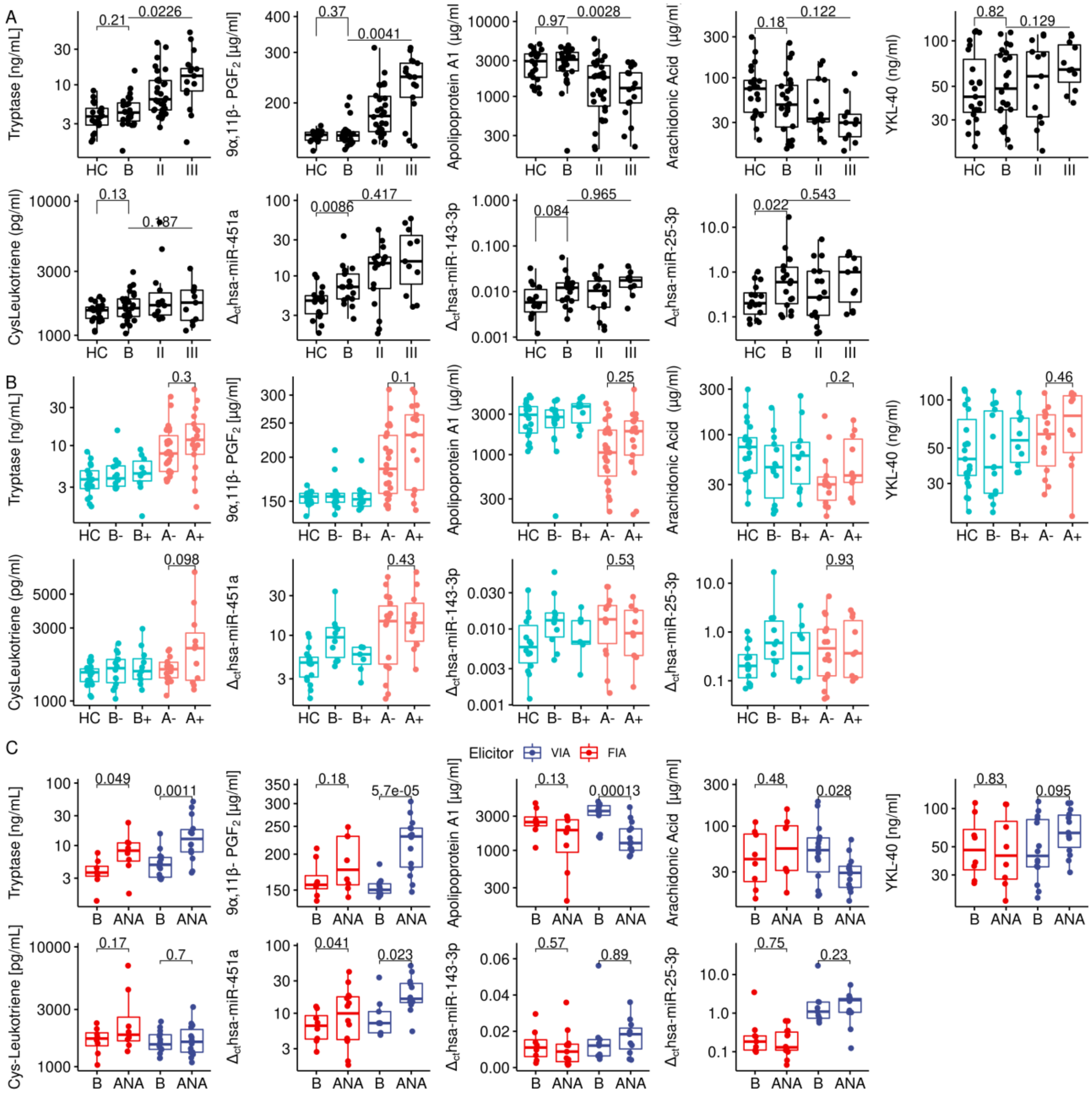

Figure S2: ***Serum levels of biomarkers depend on the severity of anaphylaxis, elicitor.*** *A) Severity of anaphylaxis (Ring & Messmer grade II, n = 30; and III, n = 17) and the biomarker levels HC - healthy controls (n = 24), B - anaphylaxis patients during baseline (n = 24). Comparisons between HC vs B - two-tailed Student't test; comparisons between B vs III - two-*

tailed Student's T test for paired data. B) Stratification of patients based on adrenaline administration during anaphylaxis (A+ n = 17, A- n = 27, red), and compared to healthy controls (HC, n = 24, blue) and baseline samples (B+ n = 10, B- n = 14, blue). Two-tailed Student's T test; + Received adrenaline, - did not receive adrenaline in the ER. C) Biomarkers of anaphylaxis during baseline and acute reaction depending on the elicitor. VIA - venom induced anaphylaxis (n = 14), FIA - food induced anaphylaxis (n = 8), two-tailed Student's T test for paired data.

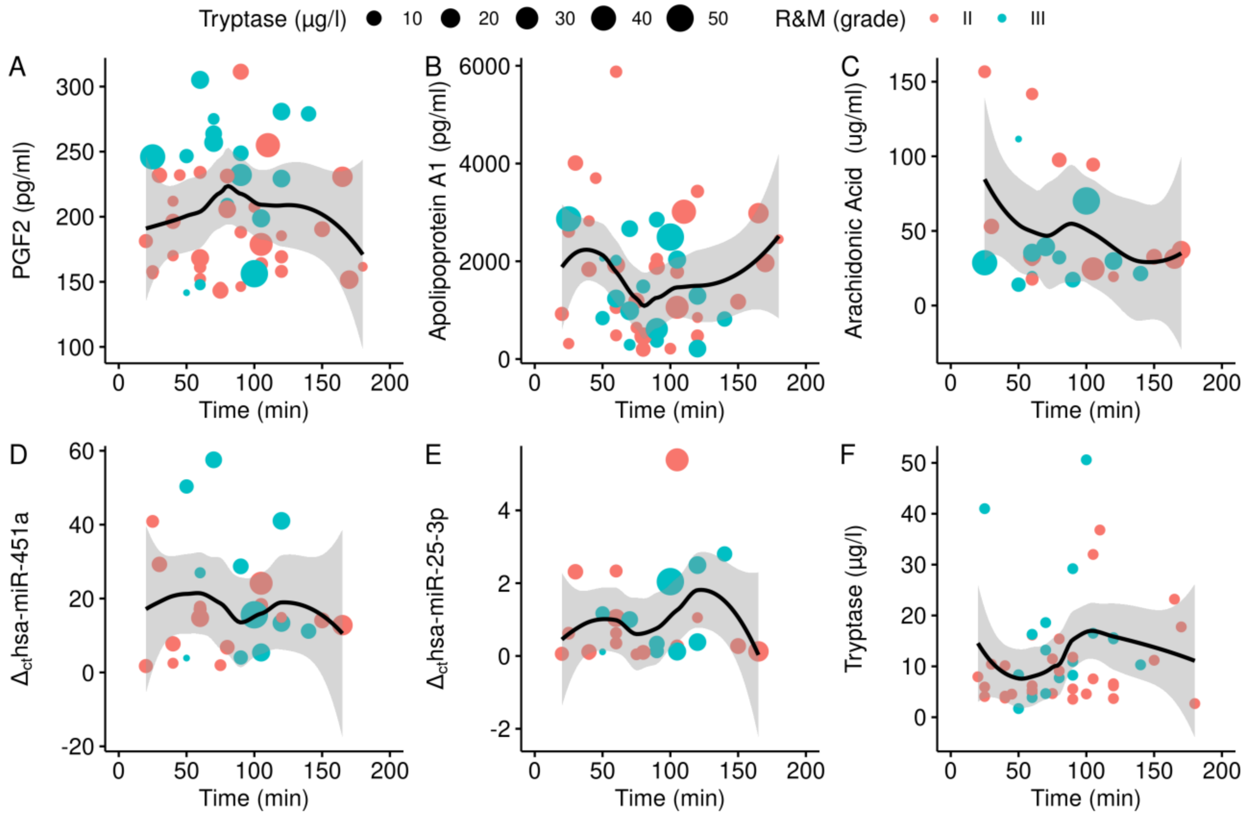

Figure S3: ***Level of biomarkers of anaphylaxis change in time from first reaction symptoms to blood draw.*** *A – F) Time between anaphylaxis onset to blood draw and its influence on the biomarkers' level. The size of the data points in the graph represents the measured serum tryptase and the color illustrates severity grade of a given episode on Ring and Messmer scale. The black line illustrates a locally estimated scatterplot smoothing (LOESS) curve (63). Grey shading represents 95% confidence intervals.*

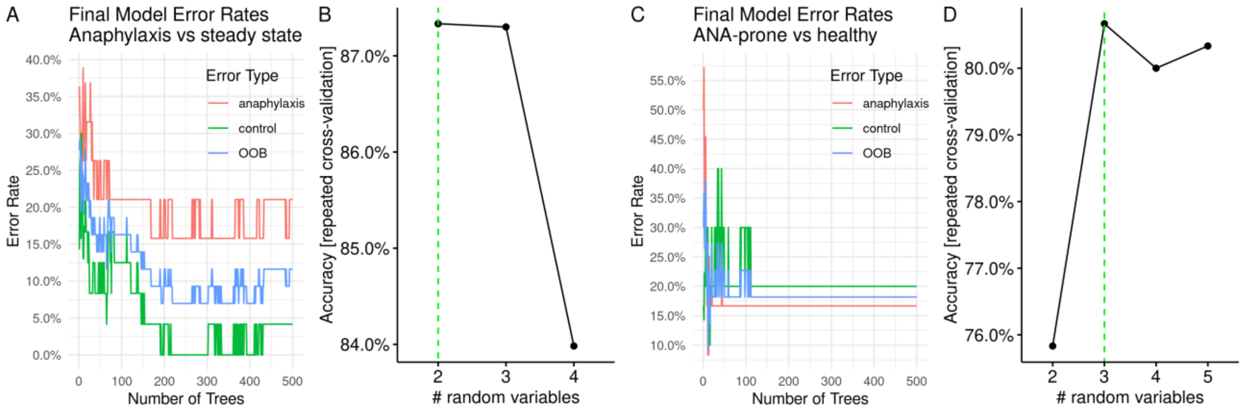

Figure S4: ***Hyperparameter selection for the random forest classifiers.*** *A – B) Anaphylaxis vs. baseline. C – D) Healthy vs. ana-prone patients (i.e. during baseline). Model error rates plotted against the number of trees grown during training. OOB - out of bag error. Model accuracy in regard to the number of randomly selected predicors upon each decision tree.*

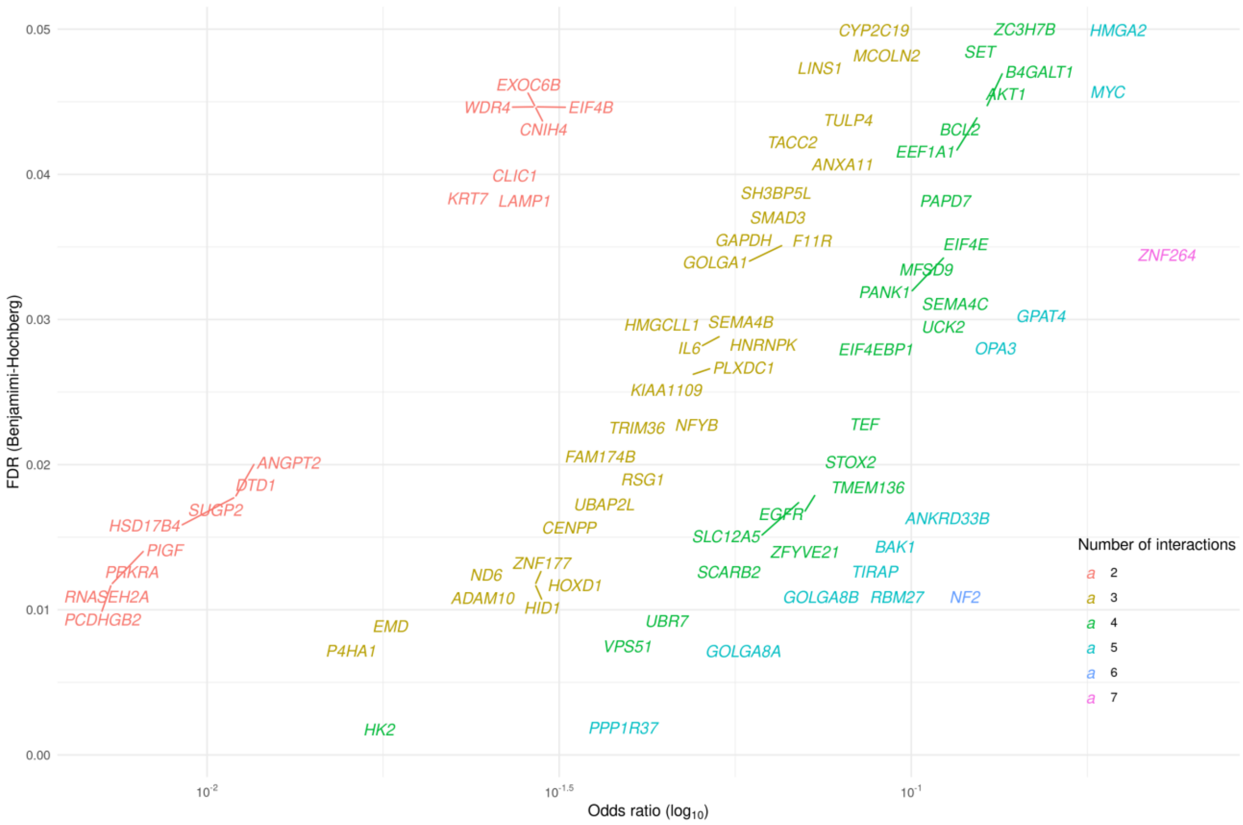

Figure S5: Predicted gene targets based on miRTarBase involving genes that were matched by at least 2 from the 19 most differentially expressed miRNAs.

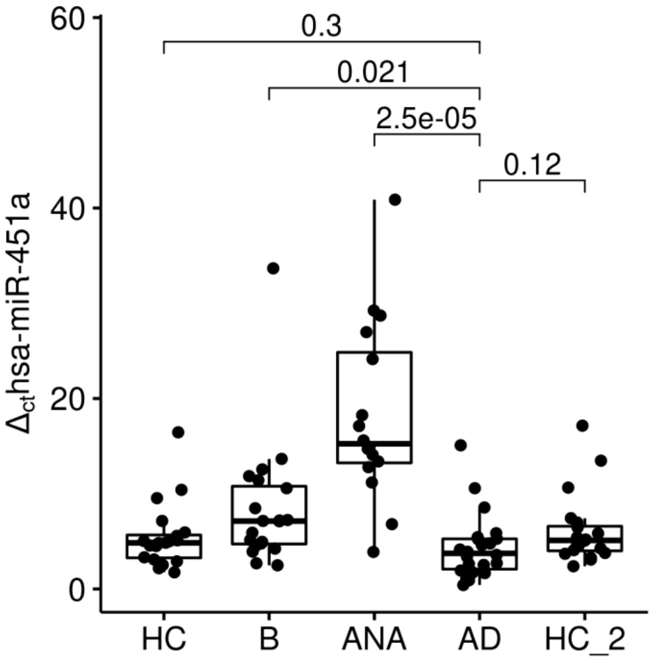

Figure S6: Comparison of hsa-mir-451a serum levels in patients undergoing anaphylaxis (ANA, n = 16), their corresponding baseline (B, n = 16) and moderate-to-severe atopic dermatitis patients (AD, n = 22) without previous history of anaphylaxis as well as two control groups (HC, n = 20 and HC_2, n = 20). Two-tailed Student's T test.

Table S1: Demographic data of the studied individuals. FIA - food induced anaphylaxis, VIA - venom induced anaphylaxis, ANA - anaphylaxis, HC - healthy control, NA - not available.

Sample ID	Age	Sex	Elicitor	Group	Ethnicity
RS01	21	female	FIA	ANA	Caucasian
RS02	26	female	FIA	ANA	Caucasian
RS08	32	female	FIA	ANA	Caucasian
RS09	24	female	DIA	ANA	Caucasian
RS10	37	male	NA	ANA	Middle East
RS12	35	female	FIA	ANA	Caucasian
RS16	29	male	FIA	ANA	Middle East
RS18	55	female	VIA	ANA	Middle East
RS22	29	female	NA	ANA	Caucasian
RS23	75	female	FIA	ANA	Caucasian
RS24	39	female	FIA	ANA	Caucasian
RS25	58	male	FIA	ANA	Caucasian
RS28	31	female	FIA	ANA	Caucasian
RS29	35	male	FIA	ANA	Caucasian
RS30	69	female	DIA	ANA	Caucasian

Sample ID	Age	Sex	Elicitor	Group	Ethnicity
RS31	37	male	VIA	ANA	Caucasian
RS32	57	female	FIA	ANA	Caucasian
RS33	77	male	VIA	ANA	Caucasian
RS34	49	male	VIA	ANA	Caucasian
RS35	53	female	VIA	ANA	Caucasian
RS36	31	female	VIA	ANA	Caucasian
RS38	62	male	VIA	ANA	Caucasian
RS42	25	female	FIA	ANA	East Asian
RS44	74	female	VIA	ANA	Caucasian
RS45	37	male	VIA	ANA	Caucasian
RS46	67	female	VIA	ANA	Caucasian
RS47	58	male	VIA	ANA	Middle East
RS48	58	male	VIA	ANA	Caucasian
RS49	50	female	VIA	ANA	Caucasian
RS51	28	male	FIA	ANA	Caucasian
RS52	53	female	VIA	ANA	Caucasian
RS53	74	male	VIA	ANA	Caucasian

Sample ID	Age	Sex	Elicitor	Group	Ethnicity
RS54	63	female	VIA	ANA	Caucasian
RS55	55	male	VIA	ANA	Caucasian
RS56	76	male	VIA	ANA	Caucasian
RS57	62	female	VIA	ANA	Caucasian
RS58	81	female	VIA	ANA	Caucasian
RS59	52	female	VIA	ANA	Middle East
RS62	54	female	VIA	ANA	Caucasian
RS63	44	male	FIA	ANA	Caucasian
RS64	64	female	FIA	ANA	Caucasian
RS65	61	male	FIA	ANA	East Asian
RS67	41	female	DIA	ANA	Caucasian
RS68	71	female	FIA	ANA	Caucasian
RS69	24	female	FIA	ANA	Caucasian
RS70	36	male	FIA	ANA	Caucasian
RS71	23	male	NA	ANA	Caucasian
KT01	51	female	NA	HC	Caucasian
KT02	29	male	NA	HC	Caucasian

Sample ID	Age	Sex	Elicitor	Group	Ethnicity
KT03	18	female	NA	HC	Caucasian
KT04	58	female	NA	HC	Caucasian
KT05	35	female	NA	HC	Caucasian
KT07	40	female	NA	HC	Caucasian
KT08	58	male	NA	HC	Caucasian
KT09	73	male	NA	HC	Caucasian
KT10	78	male	NA	HC	Caucasian
KT14	54	male	NA	HC	Caucasian
KT15	NA	male	NA	HC	Caucasian
KT19	77	female	NA	HC	Caucasian
KT20	77	male	NA	HC	Caucasian
KT21	68	female	NA	HC	Caucasian
K8	31	male	NA	HC	Caucasian
K9	31	female	NA	HC	Caucasian
K18	41	male	NA	HC	Caucasian
K19	38	female	NA	HC	Caucasian
K21	23	female	NA	HC	Caucasian

Sample ID	Age	Sex	Elicitor	Group	Ethnicity
K24	26	female	NA	HC	Caucasian
KT22	60	female	NA	HC	Caucasian
K27	31	male	NA	HC	Caucasian
K26	35	male	NA	HC	Caucasian
K25	32	male	NA	HC	Caucasian
